# The Application of Artificial Intelligence in Predicting Embryo Transfer Outcome of Recurrent Implantation Failure

**DOI:** 10.3389/fphys.2022.885661

**Published:** 2022-06-30

**Authors:** Lei Shen, Yanran Zhang, Wenfeng Chen, Xinghui Yin

**Affiliations:** ^1^ College of Computer and Information, Hohai University, Nanjing, China; ^2^ Nanjing Marine Radar Institute, Nanjing, China; ^3^ International Department of Jinling High School, Hexi Campus, Nanjing, China

**Keywords:** artificial Intelligence, RIF, embryo transfer, machine learning, IVF

## Abstract

**Background:** Recurrent implantation failure (RIF) refers to that infertile patients have undergone multiple *in vitro* fertilization (IVF) or intracytoplasmic sperm injection (ICSI) cycles and transferred multiple embryos without embryo implantation or clinical pregnancy. Due to the lack of clear evidence-based medical guidelines for the number of embryos to be transferred in RIF patients, how to obtain the highest single cycle pregnancy success rate with as few embryos transferred as possible while avoiding multiple pregnancy as much as possible, that is, how to balance the pregnancy success rate and multiple pregnancy rate, is a great challenge for clinicians and RIF patients. We urgently need an effective and reliable assisted decision-making method to help clinicians find this balance, and an artificial intelligence (AI) system will provide an efficient solution.

**Design and Methods:** In this research, we filtered out the RIF data set (*n* = 45,921) from the Human Fertilisation and Embryology Authority (HFEA) database from 2005 to 2016. The data set was divided into two groups according to the number of embryos transferred, Group A and B. Group A included 34,175 cycles with two embryos transferred, while Group B included 11,746 cycles with only one embryo transferred, each containing 44 features and a prediction label (pregnancy). Four machine learning algorithms (RF, GBDT, AdaBoost, and MLP) were used to train Group A and Group B data set respectively and 10-folder cross validation method was used to validate the models.

**Results:** The results revealed that the AdaBoost model of Group A obtained the best performance, while the GBDT model in Group B was proved to be the best model. Both models had been proved to provide accurate prediction of transfer outcome.

**Conclusion:** Our research provided a new approach for targeted and personalized treatment of RIF patients to help them achieve efficient and reliable pregnancy. And an AI-assisted decision-making system will be designed to help clinicians and RIF patients develop personalized transfer strategies, which not only guarantees efficient and reliable pregnancy, but also avoids the risk of multiple pregnancy as much as possible.

## 1 Introduction

Since the first test tube baby was born in 1978, *in vitro* fertilization-embryo transfer (IVF-ET) has become one of the most important methods to treat infertility. So far, pregnancy rate following one IVF-ET cycle can be as high as 60%. But even in the very successful units, about 10% of women undergoing *in vitro* fertilization (IVF) treatment fail repeatedly (Margalioth et al., 2006; Busnelli et al., 2020). Recurrent implantation failure (RIF) refers to that infertile patients have undergone multiple IVF or intracytoplasmic sperm injection (ICSI) cycles and transferred multiple embryos without embryo implantation or clinical pregnancy.

Currently, there is no consensus on the definition of RIF (Coughlan et al., 2014). Disputes mainly exist in the number of embryo transfer cycles, the number and quality of embryos transferred, the age of patients and so on. Stern et al. defined patients with accumulative transfer of more than 10 embryos without clinical pregnancy as RIF (Stern et al., 2003). With the development of IVF-ET technology, the total number of embryo transfer cycles and the cumulative number of embryos transferred for diagnosing RIF have also changed accordingly. At present, the definition of RIF recognized by most clinicians was proposed by Simon et al., in 2012, that is, receiving three or more embryo transfer cycles (including fresh or frozen cycles), and transferring one or two high-quality embryos each cycle without clinical pregnancy (Simon and Laufer, 2012). In addition, in 2018, Cao et al. (2018) believed that RIF can be identified as receiving ≥2 transfer cycles, with high-quality embryos in each cycle and no pregnancy. In 2021, the ESHRE survey results opinion on RIF was no more than two failed ETs (Cimadomo et al., 2021).

In order to ensure a better clinical pregnancy rate for RIF patients, the current routine transfer strategies of many reproductive centers around the world include increasing the number of embryos transferred in a single cycle to improve the success rate of single cycle pregnancy or accumulating multiple single embryo transfer (SET) cycles to obtain a final clinical pregnancy (Calhaz-Jorge et al., 2016; Penzias et al., 2017). However, these two transfer strategies have some limitations and disadvantages.

Although increasing the number of embryos transferred in a single transfer cycle can improve the clinical pregnancy rate, it will also increase the multiple pregnancy rate (Pandian et al., 2004; de Mouzon et al., 2012). Multiple pregnancy will significantly increase the possibility of maternal and infant complications, such as: miscarriage, premature birth, fetal distress, low birth weight infants, serious obstetric complications (such as postpartum hemorrhage and gestational hypertension, etc.) (Pinborg, 2005; Santana et al., 2016; Calhaz-Jorge et al., 2017). In addition, it will greatly increase the expenditure of patients and bring them a huge economic burden (Lee et al., 2016). Just as the optimal goal of assisted reproductive technologies (ART) to treat infertility should be one healthy baby at a time, multiple pregnancy is one of the most important adverse pregnancy outcomes that should be strictly prevented in this field. Therefore, improving the pregnancy success rate of RIF patients by increasing the number of embryos transferred in a single transfer cycle is actually a risk-benefit strategy, not the best one.

On the other hand, the strategy of accumulating multiple SET cycles to obtain a clinical pregnancy completely avoids the risk of multiple pregnancy caused by iatrogenic factors, but significantly increases the time cost, economic burden, and family pressure of RIF patients. For example, just as female age is closely related to pregnancy success and maternal complications, a lengthy treatment process is bound to carry great risks. The strategy is a conservative and inefficient strategy, not the best one.

Due to the lack of clear evidence-based medical guidelines for the number of embryos to be transferred in RIF patients, how to obtain the highest single cycle pregnancy success rate with as few embryos transferred as possible while avoiding multiple pregnancy as much as possible, that is, how to balance the pregnancy success rate and multiple pregnancy rate, is a great challenge for clinicians and RIF patients (Tobias et al., 2016; Kamath et al., 2020; Ma et al., 2022). We urgently need an effective and reliable assisted decision-making method to help clinicians find this balance, and the computer-aided decision-making system will provide an efficient solution.

At present, based on big data, advanced algorithms and computing power (hash rate), artificial intelligence (AI) has been rapidly developed and widely used in various fields. AI has proved its strong strength in the application of pattern recognition technologies such as speech recognition and face recognition. The connection between medical health and AI is also getting closer (Deo, 2015; Obermeyer and Emanuel, 2016). More and more applications appear in intelligent diagnosis, virtual assistant, medical imaging, drug mining, nutrition, biotechnology, hospital management, health management, mental health, wearable devices and other application scenarios. Moreover, AI, especially machine learning has rapidly demonstrated its ability to predict human fertility.

A perfect embryo transfer strategy should have at least two elements: (ⅰ) to ensure a high level of clinical pregnancy rate; (ⅱ) to reduce the multiple pregnancy rate as much as possible. For a long time, a large number of studies have been devoted to exploring appropriate embryo transfer strategies (Practice Committee of the American Society for Reproductive Medicine, 2017; Practice Committee of the American Society for Reproductive Medicine and Practice Committee of the Society for Assisted Reproductive Technology, 2017). Our aim is to use this new technology of AI to find the best embryo transfer strategy. We included the data of patients receiving ART treatment from the Human Fertilisation and Embryology Authority (HFEA), which is a world-class expert organization collecting data and statistics about the fertility treatment cycles performed each year in the United Kingdom. The data of RIF patients between 2005–2016 were filtered out to construct the data set for model training. We further divided the data set into two groups according to the embryo transfer strategy, i.e., Group A: double embryo transfer (DET) group; Group B: single embryo transfer (SET) group. Finally, two prediction models were trained and built separately using machine learning algorithms. The two models will predict the pregnancy outcome after embryo transfer (SET or DET) based on the clinical variables before embryo transfer in RIF patients who are starting a new IVF cycle. Then, the prediction results will help clinicians develop transfer strategies to achieve the best pregnancy outcome.

## 2 Materials and Methods

### 2.1 Data Set Construction

The Human Fertilisation and Embryology Authority (HFEA) is the UK’s independent regulator of fertility treatment and research using human embryos. It is a world-class expert organization in the fertility sector, and the first statutory body of this type in the world. HFEA collects data and statistics about the fertility treatment cycles performed each year in the United Kingdom to improve patient care and help researchers to conduct world-class research, whilst ensuring very strong protection of patient, donor and offspring confidentiality.

In this research, we are allowed to access a large and rich anonymized data set from HFEA. The data set cannot help to identify any patients, or children born as a result of treatment. So, no ethics approval is required for this research.

**FIGURE 1:**
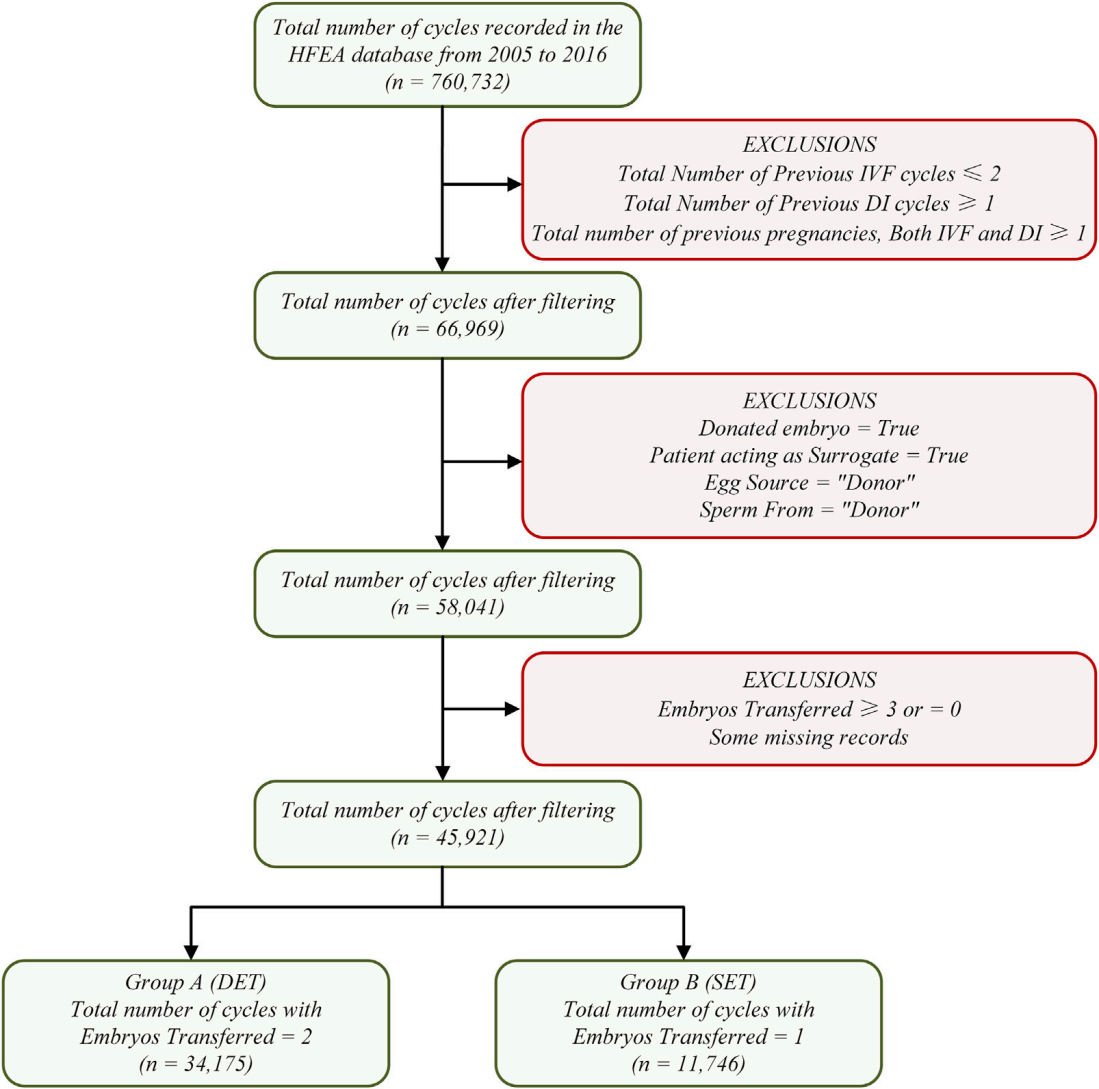
Data set construction flowchart.

The raw data set in this research contains 760,732 patient records with 95 fields on treatment cycles started between 2005 and 2016. We used more stringent criteria to filter RIF samples to avoid research errors as much as possible, that is, having received at least three transfer cycles (fresh or frozen), with high-quality embryos in each cycle, without pregnancy, and undergoing IVF treatment again (at least the fourth cycle). Data set filtering mainly includes four steps. Step 1: cycles unrelated to RIF cases were excluded. Step 2: cycles involving sperm, egg, or embryo donation, and cycles involving surrogacy were excluded. Step 3: cycles involving only one or two embryos transferred were reserved. Step 4: the remaining data were divided into two sub datasets: Group A and Group B; Group A contained 34,175 cycles with two embryos transferred and Group B contained 11,746 cycles with one embryo transferred. The entire data set filtering process is shown in Figure 1.

Focusing on the 95 fields of the raw data set, after the above data filtering steps, some fields containing exactly one value were eliminated, which are obviously meaningless to a machine learning algorithm. In addition, we also eliminated some fields that have nothing to do with RIF cases, such as fields involving donation, surrogacy, etc. Finally, 45 fields were retained. The detailed description of these 45 fields can be seen in Table 1.

**TABLE 1:** Description of 45 fields in the data set.

Field name	Field type	Description
Patient age at treatment	Categorical	Patient age at treatment, banded as follows: 18–34, 35–37, 38–39, 40–42, 43–44, 45–50
Date patient started trying to become pregnant or date of last pregnancy	Numeric	The number of years ago that patient started trying to become pregnant or years since last pregnancy
Type of infertility—female primary	Categorical	1 if the patient has never been pregnant, 0 otherwise
Type of infertility—female secondary	Categorical	1 if the patient has ever been pregnant, 0 otherwise
Type of infertility—male primary	Categorical	1 if the partner has never impregnated any woman, 0 otherwise
Type of infertility—male secondary	Categorical	1 if the partner has ever impregnated some woman, 0 otherwise
Type of infertility—couple primary	Categorical	1 if the patient has never been pregnant while the partner has never impregnated any woman, 0 otherwise
Type of infertility—couple secondary	Categorical	1 if the patient has ever been pregnant while the partner has ever impregnated some woman, 0 otherwise
Cause of infertility—tubal disease	Categorical	1 if the primary cause of infertility is due to tubal disease, 0 otherwise
Cause of infertility—ovulatory disorder	Categorical	1 if the primary cause of infertility is due to ovulatory disorder, 0 otherwise
Cause of infertility—male factor	Categorical	1 if the primary cause of infertility is due to the partner, 0 otherwise
Cause of infertility—patient unexplained	Categorical	1 if the primary cause of infertility is unknown, 0 otherwise
Cause of infertility—endometriosis	Categorical	1 if the primary cause of infertility is due to endometriosis, 0 otherwise
Cause of infertility—cervical factors	Categorical	1 if the primary cause of infertility is due to cervical factors, 0 otherwise
Cause of infertility—partner sperm concentration	Categorical	1 if the primary cause of infertility is due to partner sperm concentration, 0 otherwise
Cause of infertility—partner sperm morphology	Categorical	1 if the primary cause of infertility is due to partner sperm morphology, 0 otherwise
Cause of infertility—partner sperm motility	Categorical	1 if the primary cause of infertility is due to partner sperm motility, 0 otherwise
Cause of infertility—partner sperm immunological factors	Categorical	1 if the primary cause of infertility is due to partner sperm immunological factors, 0 otherwise
Stimulation used	Categorical	1 if this was a stimulated cycle, 0 otherwise
Specific treatment type	Categorical	The specific treatment type used in this cycle, includes IVF, ICSI.
PGD	Categorical	1 if this cycle involved the use of preimplantation genetic diagnosis, 0 otherwise
PGD treatment	Categorical	1 if this cycle would be contained in the “PGD” CaFC category on the HFEA website, 0 otherwise
PGS	Categorical	1 if this cycle involved the use of preimplantation genetic screening, 0 otherwise
PGS treatment	Categorical	1 if this cycle would be contained in the “PGS” CaFC category on the HFEA website, 0 otherwise
Elective single embryo transfer	Categorical	1 if this cycle involved the deliberate use of only one embryo, 0 otherwise
Fresh cycle	Categorical	1 if this cycle used fresh embryos, 0 otherwise
Frozen cycle	Categorical	1 if this cycle used frozen embryos, 0 otherwise
Eggs thawed	Numeric	If this cycle used frozen eggs, the number of eggs thawed
Fresh eggs collected	Numeric	The number of eggs collected in this cycle
Fresh eggs stored	Numeric	The number of eggs collected in this cycle and subsequently frozen
Total eggs mixed	Numeric	The number of eggs mixed with sperm
Eggs mixed with partner sperm	Numeric	The number of eggs mixed with sperm from the partner
Total embryos created	Numeric	The total number of embryos created in this cycle
Eggs micro-injected	Numeric	The number of eggs that were injected with sperm e.g., By ICSI.
Embryos from eggs micro-injected	Numeric	The number of embryos that were created in this cycle using ICSI.
Total embryos thawed	Numeric	If this was a frozen cycle, the total number of embryos that were thawed
Embryos transferred from eggs micro-injected	Numeric	The number of embryos transferred into the patient in this cycle that were created using ICSI.
Embryos stored for use by patient	Numeric	The number of embryos that were created in this cycle and then frozen for subsequent use by the patient
Embryos (from eggs micro-injected) stored for use by patient	Numeric	The number of embryos that were created in this cycle by injecting sperm and then frozen for subsequent use by the patient
Date of egg collection	Numeric	The number of days between egg collection and the first date provided in the series: egg collection date; egg thaw date; egg mix date; embryo thaw date; embryo transfer date
Date of egg thawing	Numeric	The number of days between egg thawing and the first date provided in the series: egg collection date; egg thaw date; egg mix date; embryo thaw date; embryo transfer date
Date of egg mixing	Numeric	The number of days between egg mixing and the first date provided in the series: egg collection date; egg thaw date; egg mix date; embryo thaw date; embryo transfer date
Date of embryo thawing	Numeric	The number of days between embryo thawing and the first date provided in the series: egg collection date; egg thaw date; egg mix date; embryo thaw date; embryo transfer date
Date of embryo transfer	Numeric	The number of days between embryo transfer and the first date provided in the series: egg collection date; egg thaw date; egg mix date; embryo thaw date; embryo transfer date
Early outcome	Categorical	1 if there was an intrauterine fetal pulsation seen due to this cycle, 0 otherwise

The field “Early Outcome” indicates whether an intrauterine fetal pulsation was found, which was used as a prediction label, or output. The remaining 44 fields were defined as features, or inputs. Finally, the data set was constructed, that is, one 34,175 × 45 matrix for Group A and one 11,746 × 45 matrix for Group B.

### 2.2 Data Cleaning

In this process, our main purpose is to correct the data, eliminate the recording errors during data acquisition and standardize the data format so that the algorithm can recognize. It includes the following steps:

(ⅰ) Standardize the data format into two categories: numeric and categorical, e.g., “Patient Age at Treatment” was converted to categorical data (18–34: ‘0’, 35–37: ‘1’, 38–39: ‘2’, 40–42: ‘3’, 43–44: ‘4’, 45–50: ‘5’), “Eggs Thawed” was converted to numeric data (range from 0 to 21), “Total Embryos Thawed” was converted to numeric data (range from 0 to 26), etc.
(ⅱ) Check for missing values and outliers, e.g., all the blanks in “PGD treatment” and “PGS Treatment” were filled with 0, outliers in “Date of Egg Mixing” were deleted, etc.
(ⅲ) Apply Z-score normalization method to make the data have a mean of 0 and a standard deviation of 1, which can weaken the influence of the value range on the model (Shiffler, 1988).

In addition, due to the imbalance of the data set, that is, the number of negative cycles is greater than that of positive cycles, we randomly sampled the negative samples to ensure that the data set used for model training is a completely balanced one, which can greatly improve the classification performance.

### 2.3 Model Building

Our main purpose is to use machine learning algorithms to build a mapping from features to labels, which is similar to a specific function. Each set of features is used as the input of the function, and then the output of the function will be classified under a specific threshold. The algorithms obtain the best threshold in multiple iterations of classification and summary. At this time, the classification result is closest to the real label. That is the entire learning process. In this research, four machine learning algorithms were used to build the classification model (prediction model).

#### 2.3.1 Random Forest

Random Forest (RF) is an averaging algorithm based on randomized decision trees (DT). The algorithm is a perturb-and-combine technique specifically designed for trees (Breiman, 1998). This means a diverse set of classifiers is created by introducing randomness in the classifier construction. The prediction of the ensemble is given as the averaged prediction of the individual classifiers (Breiman, 2001). In RF, each tree in the ensemble is built from a sample drawn with replacement (i.e., a bootstrap sample) from the training set. Furthermore, when splitting each node during the construction of a tree, the best split is found either from all input features or a random subset. The purpose of these two sources of randomness is to decrease the variance of the forest estimator. Indeed, individual decision trees typically exhibit high variance and tend to overfit. The injected randomness in forests yield decision trees with somewhat decoupled prediction errors. By taking an average of those predictions, some errors can cancel out. RF achieve a reduced variance by combining diverse trees, sometimes at the cost of a slight increase in bias. In practice the variance reduction is often significant, hence yielding an overall better model (Geurts et al., 2006).

#### 2.3.2 AdaBoost

AdaBoost is to fit a sequence of weak learners (i.e., models that are only slightly better than random guessing, such as small decision trees) on repeatedly modified versions of the data (Freund and Schapire, 1997). The predictions from all of them are then combined through a weighted majority vote (or sum) to produce the final prediction. The data modifications at each so-called boosting iteration consist of applying weights $\omega_{1},\omega_{2},\omega_{3},\ldots ,\omega_{N}$ to each of the training samples. Initially, those weights are all set to $\omega_{i}=1/N$ , so that the first step simply trains a weak learner on the original data. For each successive iteration, the sample weights are individually modified and the learning algorithm is reapplied to the reweighted data. At a given step, those training examples that were incorrectly predicted by the boosted model induced at the previous step have their weights increased, whereas the weights are decreased for those that were predicted correctly. As iterations proceed, examples that are difficult to predict receive ever-increasing influence. Each subsequent weak learner is thereby forced to concentrate on the examples that are missed by the previous ones in the sequence (Hastie et al., 2009).

#### 2.3.3 Gradient Boosted Decision Tree

Gradient Tree Boosting or Gradient Boosted Decision Tree (GBDT) is a generalization of boosting to arbitrary differentiable loss functions (Friedman, 2002). GBDT is an accurate and effective off-the-shelf procedure that can be used for both regression and classification problems in a variety of areas including Web search ranking and ecology. GBRT is an additive model whose prediction $y_{i}$ for a given input $x_{i}$ is of the following form:

$$\hat{y_{i}}=F_{M}\left(x_{i}\right)=\sum_{m=1}^{M}h_{m}\left(x_{i}\right)$$

where the $h_{m}$ are estimators called weak learners in the context of boosting. GBDT uses DT of fixed size as weak learners. Similar to other boosting algorithms, GBRT is built in a greedy fashion:

$$F_{m}\left(x\right)=F_{m-1}\left(x\right)+h_{m}\left(x\right)$$

where the newly added tree $h_{m}$ is fitted in order to minimize a sum of losses $L_{m}$ , given the previous ensemble $F_{m-1}$ :

$$h_{m}=\arg\underset{h}{\min}L_{m}=\arg\underset{h}{\min}\sum_{i=1}^{n}l\left(y_{i},F_{m-1}\left(x_{i}\right)+h\left(x_{i}\right)\right)$$

where $l\left(y_{i},F\left(x_{i}\right)\right)$ is defined as loss function. Using a first-order Taylor approximation, the value of l can be approximated as follows:

$$l\left(y_{i},F_{m-1}\left(x_{i}\right)+h_{m}\left(x_{i}\right)\right)\approx l\left(y_{i},F_{m-1}\left(x_{i}\right)\right)+h_{m}\left(x_{i}\right){\left[\frac{\partial l\left(y_{i},F\left(x_{i}\right)\right)}{\partial F\left(x_{i}\right)}\right]}_{F=F_{m-1}}$$

Then, we will denote $g_{i}$ as follows:

$$g_{i}={\left[\frac{\partial l\left(y_{i},F\left(x_{i}\right)\right)}{\partial F\left(x_{i}\right)}\right]}_{F=F_{m-1}}$$

Removing the constant terms, we have:

$$h_{m}\approx \arg\underset{h}{\min}\sum_{i=1}^{n}h\left(x_{i}\right)g_{i}$$

This is minimized if $h\left(x_{i}\right)$ is fitted to predict a value that is proportional to the negative gradient $-g_{i}$ . Therefore, at each iteration, the estimator $h_{m}$ is fitted to predict the negative gradients of the samples. The gradients are updated at each iteration. This can be considered as some kind of gradient descent in a functional space.

#### 2.3.4 Multi-layer Perceptron

Multi-layer Perceptron (MLP), also known as Artificial Neural Network (ANN), is a supervised learning algorithm that learns a function $f\left(\cdot \right):R^{m}\to R^{o}$ by training on a data set, where m is the number of dimensions for input and o is the number of dimensions for output. It can learn a non-linear function approximator for either classification or regression. It is different from logistic regression, in that between the input and the output layer, there can be one or more non-linear layers, called hidden layers. Given a set of training examples $\left(x_{1},y_{1}\right),\left(x_{2},y_{2}\right),\cdots \left(x_{n},y_{n}\right)$ where $x_{i}\in R^{n}$ and $y_{i}\in \left\{0,1\right\}$ , a one hidden layer one hidden neuron MLP learns the function $f\left(x\right)=W_{2}g\left(W_{1}^{T}x+b_{1}\right)+b_{2}$ where $W_{1}\in R^{m}$ and $W_{2},b_{1},b_{2}\in R$ are model parameters. $W_{1},W_{2}$ represent the weights of the input layer and hidden layer, respectively; and $b_{1},b_{2}$ represent the bias added to the hidden layer and the output layer, respectively. g(⋅):R→R is the activation function. For binary classification, f(x) passes through the logistic function $g\left(z\right)=1/\left(1+e^{-z}\right)$ to obtain output values between zero and one. A threshold, set to 0.5, would assign samples of outputs larger or equal 0.5 to the positive class, and the rest to the negative class. The loss function for classification is Cross-Entropy, which in binary case is given as,

$$Loss\left(\hat{y},y,W\right)=-y\ln\hat{y}-\left(1-y\right)\ln\left(1-\hat{y}\right)+\alpha \|W{\|}_{2}^{2}$$

Starting from initial random weights, MLP minimizes the loss function by repeatedly updating these weights. After computing the loss, a backward pass propagates it from the output layer to the previous layers, providing each weight parameter with an update value meant to decrease the loss. The algorithm stops when it reaches a preset maximum number of iterations; or when the improvement in loss is below a certain, small number (Rumelhart et al., 1986; Widrow and Lehr, 2002).

### 2.4 Model Validation

Hold-out method: divide the data set D into two mutually exclusive subsets, one as the training set S and the other as the test set T , i.e., D=S∪T,S∩T=∅ . After training the model on S , use T to evaluate its test error as an estimate of the generalization error. In this research, we used 70% of the data set as the training set and 30% as the test set.

K-folder cross validation method: randomly divide the data set D into K mutually exclusive subsets of the same size, i.e., $D=D_{1}\cup D_{2}\cup \ldots \cup D_{K},D_{i}\cap D_{j}=\emptyset \left(i\neq j\right)$ . Each time randomly select K−1 subsets as the training set, and the remaining one as the test set. In each of these K training and validation runs, the subset used to validate the model is different. Finally, we average the training error and validation error respectively for these K runs. In this research, K was set to 10, i.e., 10-folder cross validation (Kohavi, 1995; Rao and Fung, 2008).

We used hold-out method to pre-select the models, which can quickly eliminate some models that are not suitable for this research. 10-folder cross validation was used to conduct in-depth analysis of the model, which can make full use of the distribution characteristics of the data set.

### 2.5 Performance Measure

In machine learning research, confusion matrix is a visualization technique used to summarize the performance of classification algorithms, especially for supervised learning. In binary classification, the confusion matrix consists of four elements, which are true negatives (*TN*), true positives (*TP*), false positives (*FP*), and false negatives (*FN*). In a binary confusion matrix, upper left corner (*TN*) and lower right corner (*TP*) refer to correct classification performance; lower left corner (*FN*) and upper right (*FP*) corner refer to incorrect classification performance.

*Accuracy* is the ratio of the number of correctly predicted samples to the total number of samples, i.e.,

$$Accuracy=\frac{TP+TN}{TP+FP+TN+FN}$$

*Accuracy* seems to be a good evaluation metric, but in practice, it may hide a lot of details we need. which hinders a better understanding of the performance of the classification model. Therefore, we need to calculate more metrics from the four elements of the confusion matrix to obtain a more comprehensive performance measure (Stralen et al., 2009). At this time, we used the following metrics:

*Precision* indicates the ratio of samples correctly predicted as positive to the total samples predicted as positive, i.e.,

$$Precision=\frac{TP}{TP+FP}$$

*Recall* indicates the ratio of samples correctly predicted as positive to the total positive samples, i.e.,

$$Recall=\frac{TP}{TP+FN}$$

*Sensitivity* also represents the positive classification performance, which has the same formula as *Recall. Specificity* indicates the ratio of samples correctly predicted as negative to the total negative samples, i.e.,

$$\begin{array}{cc} Sensitivity=\frac{TP}{TP+FN} & Specificity=\frac{TN}{TN+FP} \end{array}$$

*Precision* and *Recall* are contradictory, and we cannot have them both. If both *Precision* and *Recall* are considered comprehensively, a new evaluation metric *F1-score*, also known as the comprehensive classification rate, can be obtained. The formula is as follows:

$$F1\;score=2*\frac{Precision\times Recall}{Precision+Recall}$$

The Precision-Recall curve (P-R curve) is actually a curve composed of two variables, *Precision* and *Recall*, in which the horizontal axis represents *Recall,* and the vertical axis represents *Precision.* A threshold corresponds to a point on the PR curve. By selecting an appropriate threshold, the samples are classified, and then calculate the corresponding *Precision* and *Recall*. The P-R curve can be drawn by connecting the points formed by many sets of *Precision* and *Recall* corresponding to different thresholds. The P-R curve is closer to the upper right corner (i.e., *Precision* = 1, *Recall* = 1), the better the performance of the classifier. Moreover, if the P-R curve of one classifier is completely enclosed by the P-R curve of another classifier, it can be asserted that the performance of the latter is better than that of the former (Mosley, 2013).

Receiver operating characteristic curve (ROC curve) was also used in this research. In the ROC curve, the horizontal axis is defined as false positive rate (FPR), and the vertical axis is defined as true positive rate (TPR). Similar to the P-R curve, the corresponding FPR and TPR are calculated after selecting an appropriate threshold, and the curve is drawn by connecting the points formed by different sets of FPR and TPR. The ROC curve is closer to the upper left corner (i.e., TPR = 1, FPR = 0), the better the performance of the classifier (Fawcett, 2005).

Moreover, area under receiver operating characteristic curve (AUC-ROC), which can intuitively evaluate the performance of the classifier, has a numerical value range from 0 to 1, where 1 represents the best performance, 0 is the worst, 0.5–1 means the model can predict by setting an appropriate threshold and AUC-ROC = 0.5 means no predictive value like random guessing. In the same way, we can also calculate the area under Precision-Recall curve (AUC-PR), that is, the larger the value, the better the performance (Hanley and Mcneil, 1982).

## 3 Results

### 3.1 Cycle Data Statistics

The data set included in this research was filtered from 760,732 cycle records in the HFEA database from 2005 to 2016. A total of 45,921 cycle records were used in the model training and validation process. They were divided into two groups according to the number of embryos transferred, Group A and Group B. Group A contained 34,175 cycles with two embryos transferred, while Group B contained 11,746 cycles with only one embryo transferred. “Early Outcome” (intrauterine fetal pulsation) was used as the prediction label (or output), and the remaining 44 fields were identified as features (or input). As mentioned above, the entire data set included one 34,175 × 45 matrix for Group A and one 11,746 × 45 matrix for Group B.

In Group A, the total number of cycles was 34,175, including 9,333 positive outcome cycles and 24,842 negative outcome cycles. Focusing on the feature “Patient Age at Treatment”, among all cycles, the patients aged 18–34 had the largest proportion (*n* = 12,784, 37.41%), followed by those aged 35–37 (*n* = 9,748, 28.52%). Focusing on the features “Type of Infertility”, “Couple Primary” cycles accounted for the largest proportion (*n* = 10,179, 29.78%), followed by the “Female Primary” cycles (*n* = 8,985, 26.29%) and “Male Primary” cycles (*n* = 8,699, 25.45%). Focusing on the features “Cause of Infertility”, “Male Factor” cycles accounted for the largest proportion (*n* = 13,501, 39.51%), and “Unexplained” cycles had the second largest proportion (*n* = 9,339, 27.33%). Moreover, “Stimulation used” cycles had a higher percentage (*n* = 21,936, 64.19%).

In Group B, the total number of cycles was 11,746, including 2,386 positive outcome cycles and 9,360 negative outcome cycles. The proportion of patients aged 18–34 was still the largest (*n* = 4,121, 35.08%), followed by those aged 35–37 (*n* = 3,025, 25.75%). Among the features “Type of Infertility”, “Couple Primary” cycles accounted for the largest proportion (*n* = 1844, 15.70%), followed by the “Female Primary” cycles (*n* = 1,624, 13.83%) and “Male Primary” cycles (*n* = 1,554, 13.23%). Among the features “Cause of Infertility”, “Male Factor” cycles accounted for the highest proportion (*n* = 4,340, 36.95%), and “Unexplained” cycles had the second highest proportion (*n* = 3,352, 28.54%). In this group, cycles without “Stimulation used” had a higher percentage (*n* = 6,824, 58.10%).

### 3.2 Model Evaluation and Analysis

As shown in Table 2, a total of eight metrics were used to evaluate model performance, the specific definitions of these eight metrics have been introduced above. Eight metrics were divided into two groups: one group contains five separate performance metrics, i.e., *Accuracy*, *Precision*, *Recall*, *Sensitivity*, and *Specificity*; the other group contains 3 comprehensive performance metrics, i.e., *F1-score*, AUC-ROC and AUC-PR.

**TABLE 2:** Evaluation metrics of all models.

Model	Accuracy (%)	Precision (%)	Recall (%)	Sensitivity (%)	Specificity (%)	F1-score (%)	AUC-ROC	AUC-PR
Group A (*n* = 34,175)								
RF	73.16	74.87	68.67	68.67	77.54	71.64	0.7856	0.7902
GBDT	76.02	77.85	71.85	71.85	80.08	74.73	0.8066	0.8134
AdaBoost	76.16	78.23	71.64	71.64	80.57	74.79	0.8129	0.8206
MLP	76.25	79.58	69.79	69.79	82.55	74.36	0.8139	0.8197
Group B (*n* = 11,746)								
RF	84.71	86.31	83.10	83.10	86.36	84.67	0.8954	0.9003
GBDT	85.06	85.60	84.89	84.89	85.23	85.24	0.9025	0.9043
AdaBoost	84.50	85.24	84.07	84.07	85.09	84.94	0.9031	0.9114
MLP	84.22	87.02	81.04	81.04	87.50	83.93	0.8969	0.9032

**FIGURE 2:**
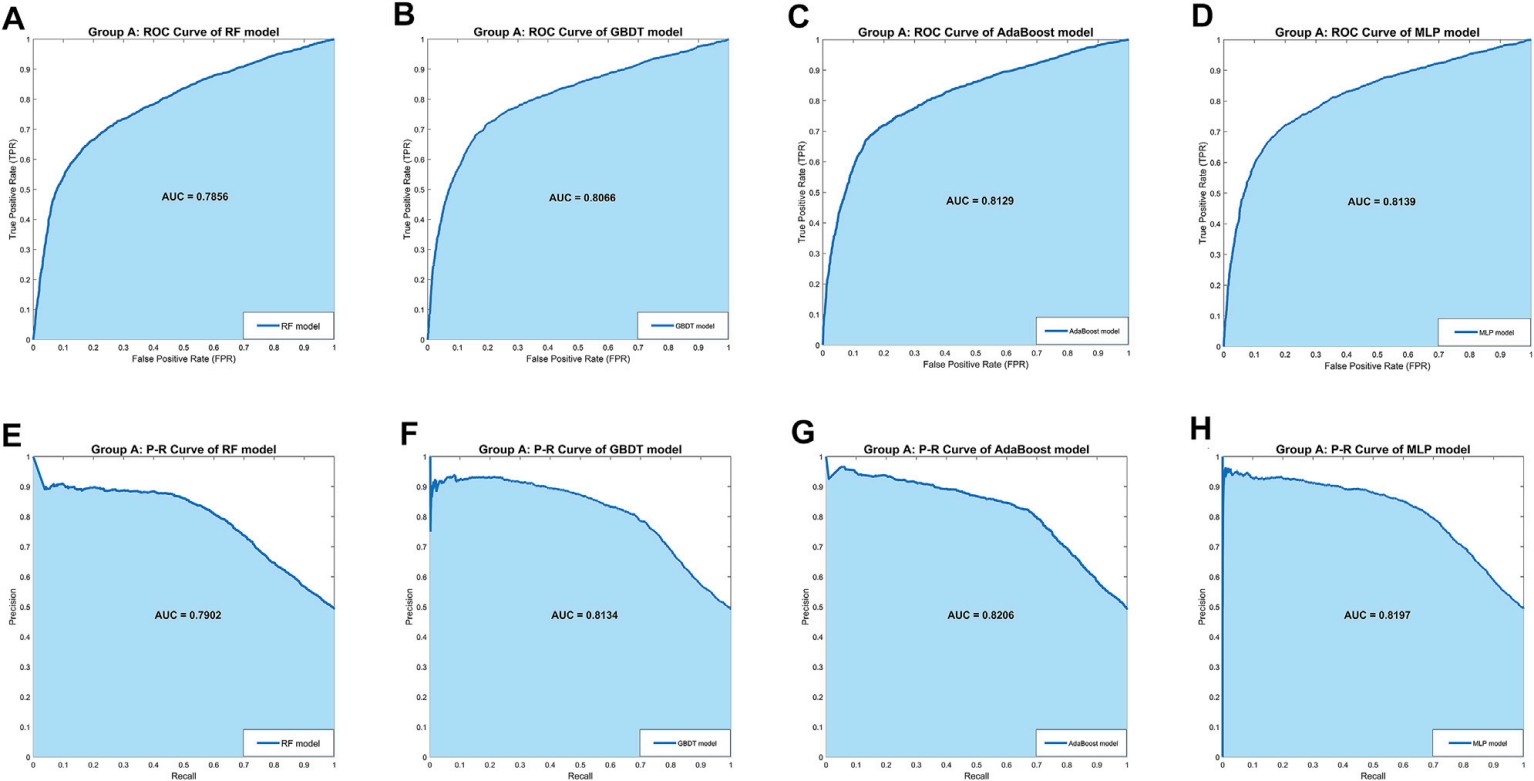
The ROC curves and P-R curves of the four models in Group A. **(A–D)** The ROC curves and AUC scores of four models in Group A: the dark blue curve refers to the ROC curve, the area under the curve is covered by light blue color and the AUC score is clearly marked. **(E–H)** The P-R curves and AUC scores of four models in Group A: the dark blue curve refers to the P-R curve, the area under the curve is covered by light blue color and the AUC score is clearly marked.

In Group A, the MLP model got the highest *Accuracy* (76.25%) and *Precision* (79.58%), but the *Recall* (69.79%) was very low. The MLP model also obtained the highest *Specificity* (82.55%) and lower *Sensitivity* (69.79%), indicating that this model was not balanced. The RF model obtained the lowest scores in all five separate performance metrics. The GBDT model and the AdaBoost model got very balanced scores in all five separate performance metrics. Among the three comprehensive performance metrics, the AdaBoost model won the best *F1-score* (74.79%) as well as AUC-PR (0.8206), and also won the second-best AUC-ROC (0.8129). Figure 2 shows the ROC curves and P-R curves of the four models in Group A respectively, and the shaded part under the curve is the AUC. In Figures 4A,B, the ROC curves and P-R curves of the four models in Group A were superimposed together and the green curves (AdaBoost model) wrapped more of the other curves. After comprehensive comparison, we found that the AdaBoost model was the model with the most balanced performance, that is, the best model.

**FIGURE 3:**
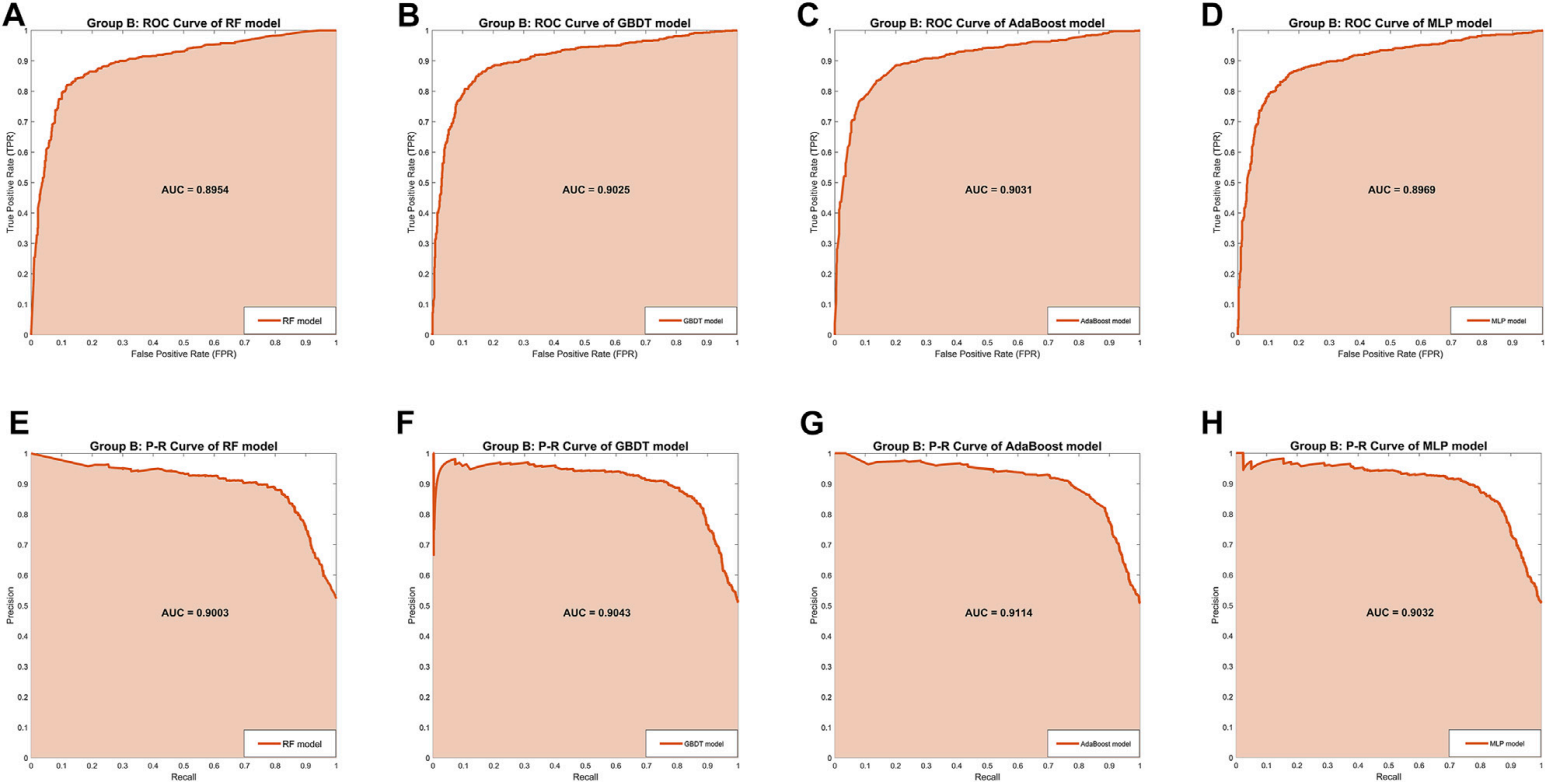
The ROC curves and P-R curves of the four models in Group B. **(A–D)** The ROC curves and AUC scores of four models in Group B: the dark orange curve refers to the ROC curve, the area under the curve is covered by light orange color and the AUC score is clearly marked. **(E–H)** The P-R curves and AUC scores of four models in Group A: the dark orange curve refers to the P-R curve, the area under the curve is covered by light orange color and the AUC score is clearly marked.

**FIGURE 4:**
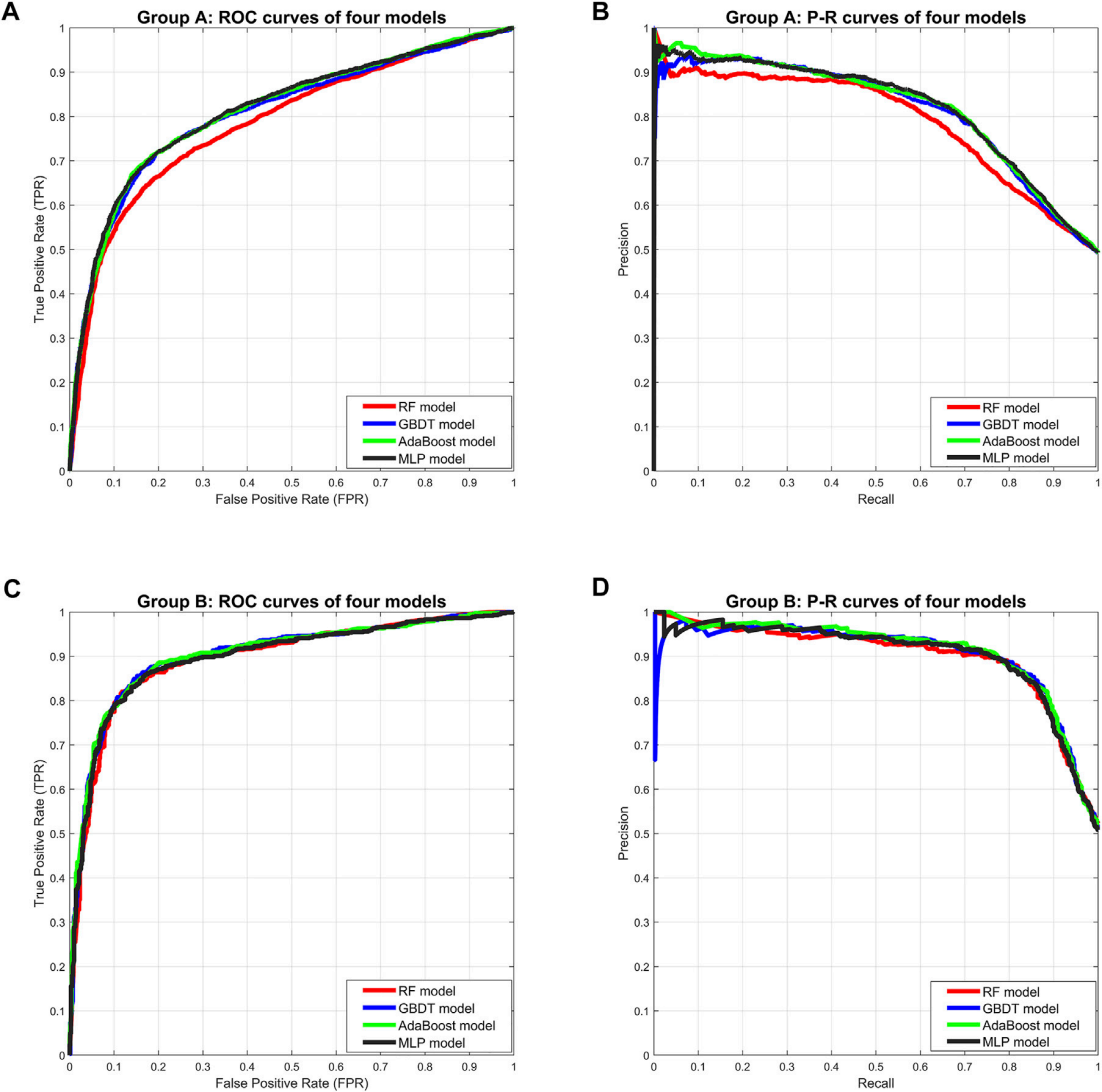
Comprehensive comparison of four models. **(A)** Comprehensive ROC curves of four models in Group A: the larger the area under the curve, the better the performance. **(B)** Comprehensive P-R curves of four models in Group A: the larger the area under the curve, the better the performance. **(C)** Comprehensive ROC curves of four models in Group B. **(D)** Comprehensive P-R curves of four models in Group B.

In Group B, the MLP model obtained the highest *Precision* (87.02%) and *Specificity* (87.50%), along with the lowest *Recall* (81.04%) and *Sensitivity* (81.04%), indicating that this model was still unbalanced in this group. The GBDT model obtained the best *Accuracy* (85.06%) and the RF model got the second-best *Accuracy* (84.71%). But in the other four separate performance metrics, the imbalance of the RF model was slightly larger than the GBDT model and the AdaBoost model, while the GBDT model was slightly better than the AdaBoost model. We turned our focus to the comprehensive performance metrics, where the GBDT model won the best *F1-score* (85.24%), but the AdaBoost model won the best AUC-ROC (0.9031) and AUC-PR (0.9114). In addition, Figure 3 shows more details of the curves and their shadow areas. In Figures 4C,D, the ROC curves and P-R curves of the four models in Group B were superimposed together and the green and blue curves (AdaBoost model and GBDT model) wrapped more of the other curves. The metrics and curves proved that the GBDT model obtained the best classification performance in Group B, that is, the best classifier.

Although we strictly evaluated the models according to the metrics and curves, in fact, the performance difference of these four models is not large, especially the GBDT model and AdaBoost model.

## 4 Discussion

RIF patients, as a special group in the infertile population, are still unable to obtain pregnancy and live birth after multiple IVF-ET cycles. At present, it is believed that a personalized embryo transfer strategy may be the most direct and effective method to improve the success rate of embryo transfer and obtain a good pregnancy outcome (Patel et al., 2019). To ensure clinical pregnancy rates in RIF patients, transfer strategies in many reproductive centers include: (ⅰ) increasing the number of embryos transferred in a single cycle; (ⅱ) accumulating multiple single embryo transfer (SET) cycles. As we mentioned above, both strategies have limitations and disadvantages: the former will significantly increase the multiple pregnancy rate and the risk of pregnancy; the latter will significantly increase the time cost and economic burden, as well as the patients’ mental stress. However, it is currently impossible to carry out targeted operations and treatments for each individual or even each embryo and more often, we only rely on the experience of clinicians. Obviously, we urgently need a reliable, accurate and intelligent assisted decision-making method to help clinicians and RIF patients develop personalized transfer strategies, which not only guarantees efficient and reliable pregnancy, but also avoids the risk of multiple pregnancy as much as possible.

In this research, we focused on AI. AI is a new technical science that studies and develops theories, methods, technologies and application systems for simulating, extending and expanding human intelligence. AI is a branch of computer science that attempts to understand the essence of intelligence and produce a new type of intelligent machine that responds in a similar way to human intelligence. Research in this field includes robotics, language recognition, image recognition, natural language processing and expert systems, etc. Since the birth of AI, theories and technologies have become increasingly mature, and the application fields have been expanding. Currently, AI, especially machine learning has rapidly demonstrated its ability to predict human fertility (Curchoe and Bormann, 2019). The research indicated that machine learning models including patients’ data can assist clinicians to offering couples undergoing IVF personalized treatment strategies (Siristatidis et al., 2016). Barnett-Itzhaki et al. tested two machine learning algorithms, SVM and artificial neural network (ANN) to predict IVF outcomes (β-HCG, clinical pregnancies, live births, etc.) (Barnett-Itzhaki et al., 2020). Raef et al. examined six machine learning approaches based on the dataset containing 82 features of IVF cycles to predict outcome and concluded that random forest (RF) was the best classifier (Raef et al., 2020). Tran et al. created a deep learning model to predict the probability of pregnancy with fetal heart (FH) from time-lapse videos (Tran et al., 2019).

In this research, we filtered out the RIF data set (*n* = 45,921) from HFEA database containing 760,732 cycle records from 2005 to 2016, and far exceeding the currently known data set of RIF patients in other studies. We divided the data set into Group A (DET, *n* = 34,175) and Group B (SET, *n* = 11,746), each containing 44 features and a prediction label (pregnancy). Four machine learning algorithms (RF, GBDT, AdaBoost, and MLP) were used to train Group A and Group B data set respectively and 10-folder cross validation method was used to validate the models. The results revealed that the AdaBoost model of Group A obtained the best performance, while the GBDT model in Group B was proved to be the best model. Both models had been proved to provide accurate prediction of transfer outcome. Naturally, our research still has some limitations. Our data are all from the United Kingdom and are limited in terms of ethnic and geographic diversity. Some potential specific indicators may not be fully covered. In addition, natural-cycle IVF (NC-IVF) and stimulated IVF (SIVF) have a different basis for success. SET in NC-IVF, which is most commonly as being used in poor responders, and SET in SIVF, which is most commonly used in high responders, have a different basis. This factor should be taken into account when setting up future models.

In conclusion, our research provided a new approach for targeted and personalized treatment of RIF patients to help them achieve efficient and reliable pregnancy. In addition, As we first imagined, an AI-assisted decision-making system is being designed for clinical application. The clinical variables before embryo transfer stage of RIF patients undergoing a new cycle will be entered into the system. The date of embryo transfer (cleavage embryo or blastocyst) as an unknown variable can be finely set as an input option. Then, the two prediction models in the system start to work separately, and prediction results will be calculated, that is, results after SET and DET. When the success rate of SET is predicted to be high, the system suggests that SET is preferred to reduce the multiple pregnancy rate. When the predicted success rate of SET is much lower than that of DET, clinicians can choose whether to perform DET according to the patient’s own wishes. The AI-assisted decision-making system cannot replace the decision-making of clinicians. It only provides suggestions. Furthermore, we will also open the model upgrade interface. More samples or features can be incorporated for training with the goal to improve the prediction accuracy and to make the prediction results more multidimensional (not just predicting SET and DET).

## Data Availability

The datasets presented in this study can be found in online repositories. The names of the repository/repositories and accession number(s) can be found below: https://www.hfea.gov.uk/about-us/our-data/#ar.
